# A 13.42-kb tandem duplication at the *ASIP* locus is strongly associated with the depigmentation phenotype of non-classic Swiss markings in goats

**DOI:** 10.1186/s12864-022-08672-9

**Published:** 2022-06-13

**Authors:** Jiazhong Guo, Xueliang Sun, Ayi Mao, Haifeng Liu, Siyuan Zhan, Li Li, Tao Zhong, Linjie Wang, Jiaxue Cao, George E. Liu, Hongping Zhang

**Affiliations:** 1 College of Animal Science and Technology, Sichuan Agricultural University, Chengdu, 611130 China; 2 Animal Genomics and Improvement Laboratory, BARC, Agricultural Research Service, USDA, Beltsville, MD 20705 USA

**Keywords:** Goat, Coat color, Swiss markings, Whole-genome sequencing, Genome-wide association study, *ASIP*, Copy number variations

## Abstract

**Background:**

The pigmentation phenotype diversity is rich in domestic goats, and identification of the genetic loci affecting coat color in goats has long been of interest. Via the detections of selection signatures, a duplication upstream *ASIP* was previously reported to be a variant affecting the Swiss markings depigmentation phenotype in goats.

**Results:**

We conducted a genome-wide association study using whole-genome sequencing (WGS) data to identify the genetic loci and causal variants affecting the pigmentation phenotype in 65 Jintang black (JT) goats (i.e., 48 solid black vs. 17 non-classic Swiss markings). Although a single association peak harboring the *ASIP* gene at 52,619,845–72,176,538 bp on chromosome 13 was obtained using a linear mixed model approach, all the SNPs and indels in this region were excluded as causal variants for the pigmentation phenotype. We then found that all 17 individuals with non-classic Swiss markings carried a 13,420-bp duplication (CHI13:63,129,198–63,142,617 bp) nearly 101 kb upstream of *ASIP*, and this variant was strongly associated (*P* = 1.48 × 10^− 12^) with the coat color in the 65 JT goats. The copy numbers obtained from the WGS data also showed that the duplication was present in all 53 goats from three European breeds with Swiss markings and absent in 45 of 51 non-Swiss markings goats from four other breeds and 21 Bezoars, which was further validated in 314 samples from seven populations based on PCR amplification. The copy numbers of the duplication vary in different goat breeds with Swiss markings, indicating a threshold effect instead of a dose-response effect at the molecular level. Furthermore, breakpoint flanking repeat analysis revealed that the duplication was likely to be a result of the Bov-B-mediated nonallelic homologous recombination.

**Conclusion:**

We confirmed that a genomic region harboring the *ASIP* gene is a major locus affecting the coat color phenotype of Swiss markings in goats. Although the molecular genetic mechanisms remain unsolved, the 13,420-bp duplication upstream of *ASIP* is a necessary but not sufficient condition for this phenotype in goats. Moreover, the variations in the copy number of the duplication across different goat breeds do not lead to phenotypic heterogeneity.

**Supplementary Information:**

The online version contains supplementary material available at 10.1186/s12864-022-08672-9.

## Introduction

Identification of the genetic loci affecting coat color in domestic goats has long been of great interest because the coat color is not only a breed-defining trait but also an economically important trait that is associated with hair fibers in goats [1]. Although the *ASIP* and *MC1R* genes are the most common regulators underlying the synthesis of black/brown eumelanin and yellow/red pheomelanin in animals (e.g., pigs [2–4], sheep [5], dogs [6, 7], chickens [8], and quails [9]), it is still challenging to pinpoint the causal variants for black coat color and other pigmentation phenotypes in goats. Strikingly, Adalsteinsson et al. proposed up to 11 different alleles at the *ASIP* locus in goats through genetic analyses of the coat color patterns in 216 goat kids and their parents [10], indicating the sequence complexity of this locus in goats. Based on genome-wide association or selection mapping analyses, previous studies revealed that the genomic region encompassing *ASIP* is a major genetic locus responsible for black/brown and yellow/red coat colors in different goat breeds [1, 11–13], but the causal variants are still unknown. Although one missense SNP (c.383G > T; p.Gly128Val) in the *ASIP* gene is found in yellow-coated breeds from Italy [14] and China [15], its allele frequencies across different populations do not suggest that this missense mutation is a plausible causal variant.

It has been demonstrated that copy number variations (CNVs) at the *ASIP* locus are the causal variants underlying coat color phenotypes in a variety of animal species, such as dogs [6], cattle [16], sheep [17], and buffaloes [18]. Recent studies identified multiple distinct CNVs surrounding or harboring the *ASIP* gene in several goat breeds, mainly from Europe [19, 20]. Using solely low pooled heterozygosity values as selection signatures, an *ASIP* allele (*A*^*sm*^) with eight tandem copies of a 13,433 bp sequence was found to control the Swiss markings phenotype in British Alpine (BAL), Toggenburg (TOG), and Grisons Striped (BST) goats. Such phenotypes are characterized by predominantly black (e.g., BAL) or brown (e.g., TOG) with white markings down the face coupled with a white belly and legs. Nevertheless, the copy numbers of the 13,433-bp sequence were just the average values across several individuals within a population, obtained from pooled whole-genome sequencing (WGS) data. Therefore, whether the copy number varies between individuals within these breeds remains elusive. Another relevant question that deserves to explore is whether variations in copy number may contribute to heterogeneity in pigmentation phenotypes of goats. Taken together, a lack of compelling evidence has left the genetic basis of the Swiss marking phenotype in goats less well understood.

Since 2018, 10 goats (five male and five female) having the similar coat color patterns to those of BAL (i.e., tan or beige coat colors on a black background, hereafter referred to as non-classic Swiss markings for the sake of simplicity) were collected from farmers at Jintang county (Chengdu, China) and raised on a Jintang Black goat farm. These goats with non-classic Swiss markings then served as parents to mate with Jintang Black goats (JT, a solid black breed). We further observed that the non-classic Swiss marking depigmentation phenotype is dominant to the solid black coat color in this population, which produced a valuable resource population for the genetic analyses of coat color in goats. As a proof of principle, here we performed a genome-wide association study using high-density genotypes from WGS data for 65 JT goats to identify the genetic loci responsible for the non-classic Swiss marking depigmentation phenotype in goats.

## Results

### Detection of short variants and population structure analysis

The alignment of short-read WGS data against the goat reference genome (i.e., the ARS1 assembly) generated an average sequencing depth of 6.06× (ranging from 5.28× to 8.69×) and genome coverage of 98.74% across 65 JT goats (Additional file 1). A total of 14,531,193 single nucleotide polymorphisms (SNPs) (14,462,675 biallelic and 68,518 multiallelic) and 1,314,832 short insertions and deletions (indels) were identified across the goat autosomal genome.

The principal component analysis (PCA) showed that the 65 JT goats were divided into two groups based on the first two PCs (Additional file 2), generally consistent with the classification according to the coat color (48 solid black goats vs.17 JT goats with non-classic Swiss markings). However, the proportion of the total genetic variance explained by the first two PCs (4.78 and 3.91%) was low. The low genome-wide average *F*_ST_ value (weighted mean *F*_ST_ = 0.037) also revealed no substantial genetic differentiation between the two groups. Therefore, the population stratification was not a major confounding factor in our GWAS samples.

### A major locus affecting non-classic Swiss markings mapped to a genomic region encompassing *ASIP* on chromosome 13

**Fig. 1:**
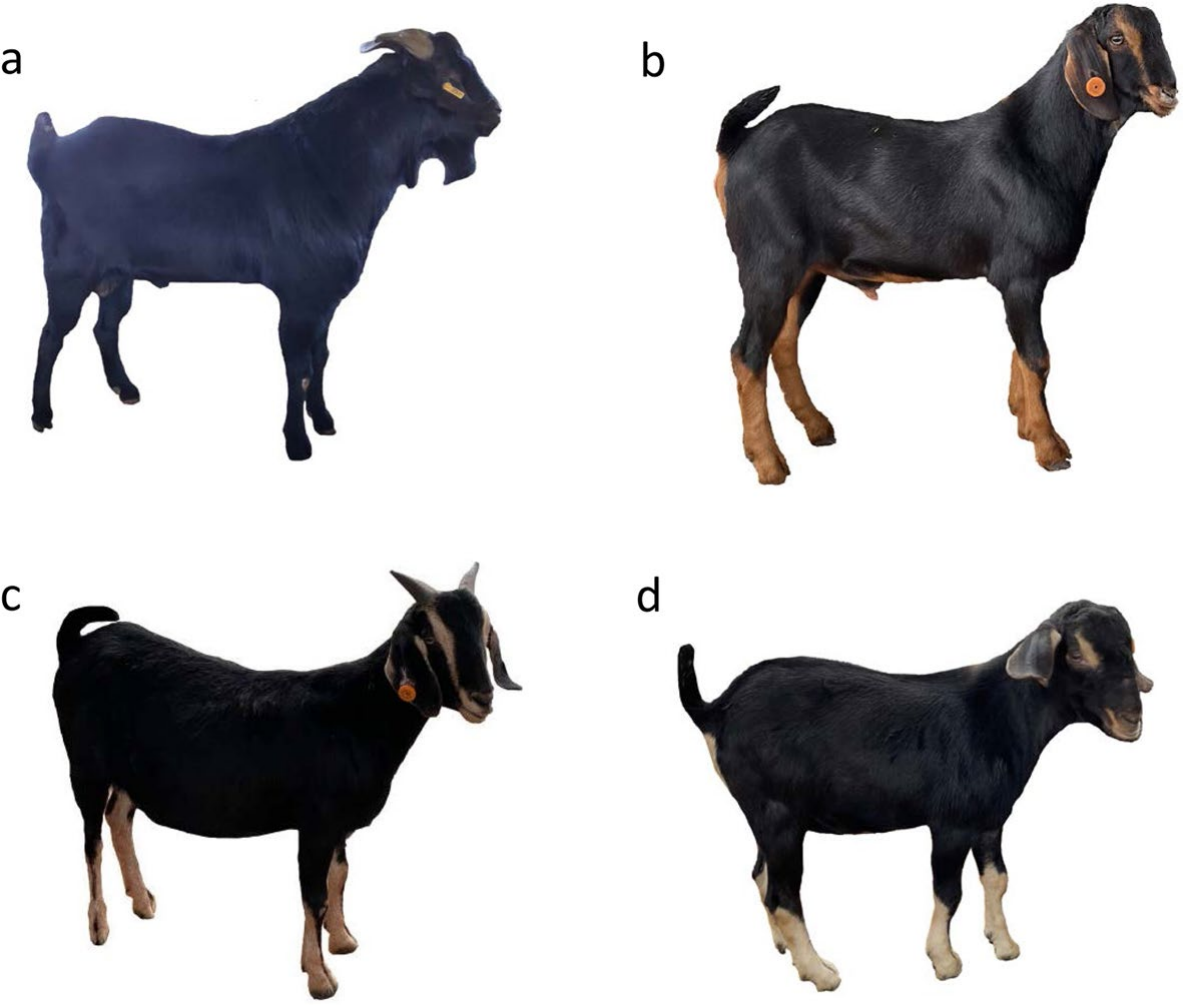
Photos of the representative coat color phenotypes in JT goats investigated in this study. A solid black JT goat is shown in **a**. **b–d** show the JT goats having tan markings, beige markings, and beige points on the face, respectively. The photo of the goats having tan points on the face was unavailable

**Fig. 2:**
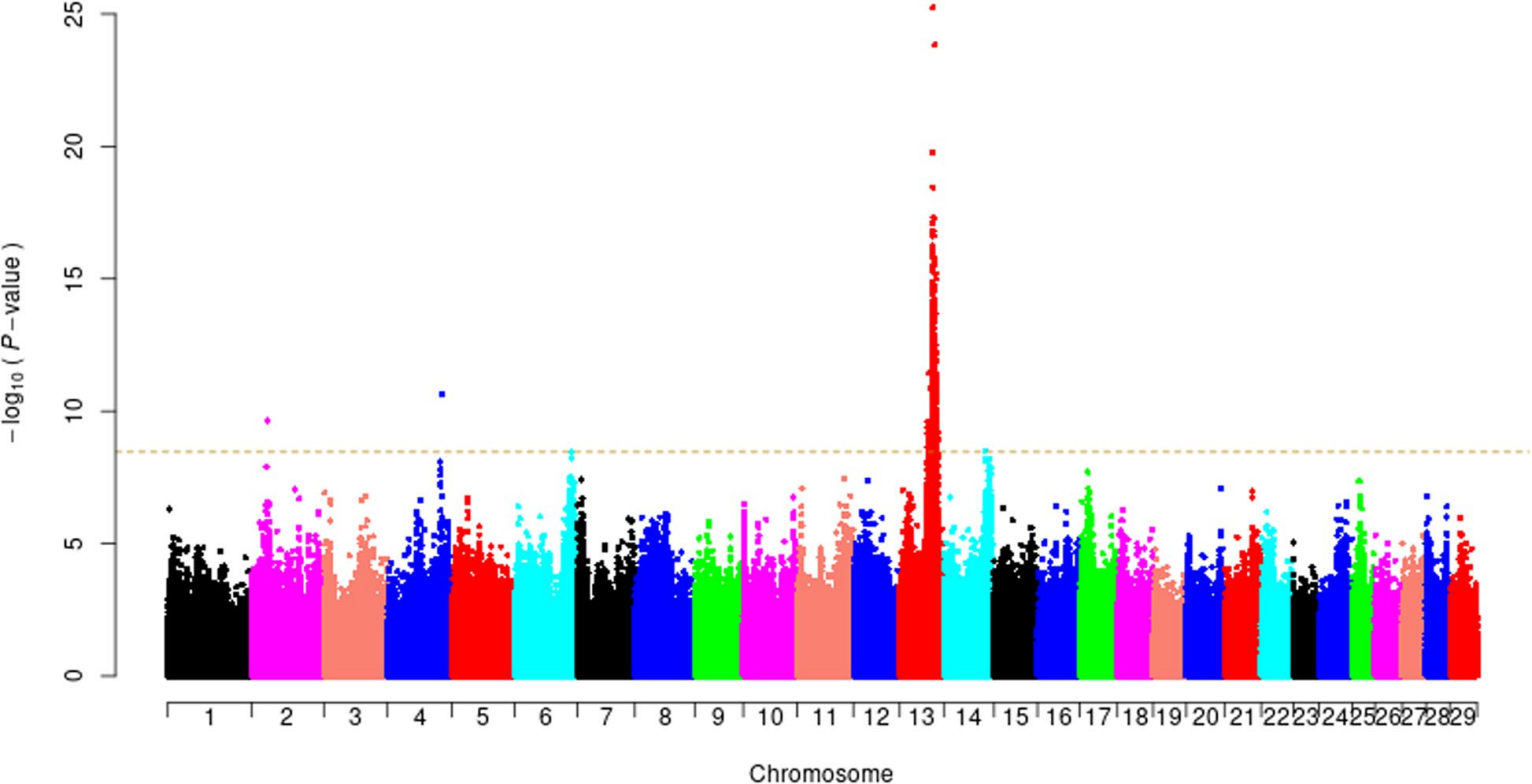
Genome-wide association study for the non-classic Swiss marking pigmentation phenotype in 65 JT goats based on a linear mixed model and genomic control. The Manhattan plot shows the association results of 14,412,316 biallelic SNPs on autosomes 1–29 with the coat color phenotype in 65 JT goats (48 solid black vs. 17 non-classic Swiss markings). The chromosomes are plotted in different colors. The *P*-values were obtained by using a mixed-model association approach and the genomic control and then transformed to –log10 (*P*-values). The horizontal dashed line indicates the genome-wide Bonferroni-corrected significance level

Although there are small phenotypical variations in 17 goats with the non-classic Swiss marking depigmentation (Fig. 1 and Additional file 1), these goats were grouped as one category in the GWAS. After the Hardy-Weinberg test (Fisher’s exact test, *P*-value > 10^− 10^) in PLINK, 14,412,316 biallelic SNPs on all 29 autosomes remained for the GWAS in 65 JT goats (48 solid black vs. 17 non-classic Swiss markings). A mixed-model association analysis with the subsequent genomic control identified a total of 660 genome-wide significantly associated SNPs (F-test, Bonferroni-corrected *P* < 0.05, −log_10_*P* = 8.46, λ = 1.09), of which 656 were located at 52,619,845–72,176,538 bp on chromosome 13 and clustered into one association peak (Fig. 2 and Additional file 3). At the top associated SNP (CHI13:62,567,187 bp, *P* = 5.69 × 10^− 26^) site, all the 48 solid black goats were homozygous for the reference allele (the ARS1 genome assembly is generated from a San Clemente goat without Swiss markings). In contrast, all animals with non-classic Swiss markings were heterozygous or homozygous for the mutant allele. According to genome annotations, this major association region encompasses 359 genes (294 protein-coding genes and 65 RNA genes), such as *RALY*, *ASIP* (CHI13:63,244,165–63,249,361 bp), *AHCY*, and *ITCH* (Additional file 4).

### None of the short variants within the major association region were the plausible causal variants for the non-classic Swiss marking phenotype

We first investigated whether the SNPs or indels were the causal variants underlying the non-classic Swiss marking phenotype in goats. There were 87,041 SNPs and 8100 indels within the major association region on chromosome 13. The variant annotation revealed that the top associated SNP was located in intron 1 of *CBFA2T2* (ENSCHIG00000017263) and was a variant with a modifier functional impact. We further examined the causality of the missense variants and the high functional impact variants using SnpEff. The variant annotations predicted 407 missense SNPs and three SNPs with a high impact. Five of them showed genome-wide associations and were located within the *ZBP1* (ENSCHIG00000006747, c.1019G > A|p.Arg340His, CHI13: 57866515 bp), *BPIFB3* (c.589G > A|p.Val197Met, CHI13: 61687255 bp), *ENSCHIG00000021281* (c.94G > A|p.Val32Ile, CHI13:61943452 bp), *RPS6 pseudogene* (ENSCHIG00000001593, c.91C > T|p.Arg31Cys, CHI13:61943478 bp), and *SOGA1* (ENSCHIG00000025181, c.2969G > C|p.Gly990Ala, CHI13:65412552 bp) genes, respectively. Apart from the leading associated SNP, nevertheless, the genotypes obtained from WGS data in three European breeds with Swiss markings (i.e., 5 BAL, 24 TOG, and 24 BST goats) and four other breeds without Swiss markings (15 Chengdu Brown [CB], 14 Tibetan Cashmere [TC], 14 Moroccan Draa [MD], and 8 Moroccan Northern [MN] goats) did not support the significant associations of the five missense SNPs with the phenotype (Additional file 5). Furthermore, only one indel (CHI13:53,790,832 bp) within the *GID8* gene showed a high functional impact, but there was no significant association between this site and the phenotype in JT goats (Fisher’s exact test, *P* = 0.84; Additional file 5). Thus, all short variants within the major association region were excluded as the causal mutations for the non-classic Swiss marking phenotype based on the genotypes in multiple breeds and the biological functions of the corresponding genes.

### A 13,420-bp tandem duplication upstream of *ASIP* is strongly associated with the non-classic Swiss marking phenotype

To find the causal mutations underlying the non-classic Swiss marking phenotype, we also conducted the genome-wide CNV calling using the WGS data in the 65 JT animals and 104 domestic goats from seven other breeds, as well as 21 Bezoars. A total of 49 raw CNVs were identified in the major association region on chromosome 13 via a read-depth approach with 1-kb sliding windows. Although one complex CNV (i.e., duplication and deletion within the same region from different individuals, CHI13:56,798,001–56,802,500 bp) were significantly associated (Fisher’s exact test, *P* = 0.03) with the coat color in 65 JT goats, the genotypes at this site in other populations did not further support their correlation (Additional file 6).

**Fig. 3:**
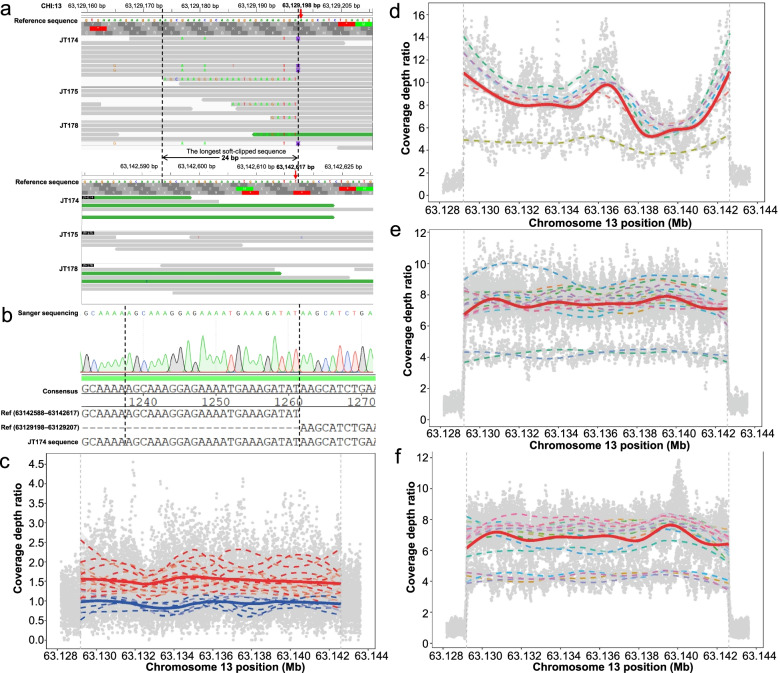
Characterization and genome coverage analysis of the 13,420-bp tandem duplication in the major associated region in JT goats and three European breeds with Swiss markings. **a** The visualization of several soft-clipped reads from WGS data reveals the 13,420-bp tandem duplication (CHI13:63,129,198-63,142,617 bp) in JT goats with non-classic Swiss markings using IGV. **b** PCR amplification and Sanger sequencing validated the presence of the duplication. **c** Genomic coverage at the duplication site in different phenotype groups of 24 JT goats (seven solid black goats vs. 17 goats with non-classic Swiss markings; adjusted for the genome-wide average coverage depth and calculated in 20-bp sliding windows). The smooth lines obtained with LOESS regressions are added to describe the trends of relative genome coverage depth along the genomic positions within each phenotype group. The same plots are shown for 5 BAL (**d**), 24 BST (**e**), and 24 TOG goats (**f**), respectively

Strikingly, the contingency table analyses revealed that one duplication (CHI13:63,128,501-63,142,500 bp) was highly associated with the coat color in the 65 JT goats (Fisher’s exact test, *P* = 1.48 × 10^− 12^). Specifically, 17 JT animals with non-classic Swiss markings were heterozygous (*n* = 14) or homozygous (*n* = 3) for the duplication (i.e., the mutant allele), whereas all the 48 solid black JT animals were homozygous for the reference allele except two goats (i.e., JT39 and JT48) (Additional file 6). Notably, none of the biallelic SNPs within the duplication showed significant associations as described above (Additional file 3). We then used the alignment information of soft-clipped reads, a class of partially mapped reads, to pinpoint the breakpoint position of the duplication. The duplication was tandem, and its exact length was 13,420 bp (CHI13:63,129,198–63,142,617 bp) (Fig. 3a and Additional file 7) in JT goats with non-classic Swiss markings, validated by PCR amplification and Sanger sequencing (Fig. 3b). The coverage depth analyses showed the sequence only duplicated once in the genomes of JT goats with non-classic Swiss markings (Fig. 3c).

Among the 104 domestic goats from seven other breeds, 53 European goats with Swiss markings (i.e., 5 BAL, 24 TOG, and 24 BST goats) all carried the same duplication (i.e., the same genomic coordinates) as those in JT goats (Fig. 3e, d, and f; Additional file 6 and Additional file 7). Strikingly, they carried more than two copies of the sequence on chromosome 13 based on the initial prediction using the coverage depth reported by the CNVcaller. The further analyses revealed that the average coverage depth of the duplication sequence was 4.10–9.31 folds of the average whole-genome coverage depth across individuals (Fig. 3e, d, and f; Additional file 8). Therefore, the genotypes at the duplication site in the 53 European goats were predicted to have two alleles: one allele—four copies of the duplication on chromosome 13, another allele—eight copies of the duplication on chromosome 13 (Additional file 8). By contrast, 45 of 51 domestic goats without Swiss markings (i.e., 15 CB, 14 TC, 14 MD, and 8 MN goats) were homozygous for the wild-type allele without duplication (Additional file 6). Furthermore, all of the 21 Bezoars (Bezoars do not have Swiss markings to our knowledge) carried the ancestral wild-type allele, implying that this duplication was a derived allele after domestication (Additional file 6).

**Fig. 4:**
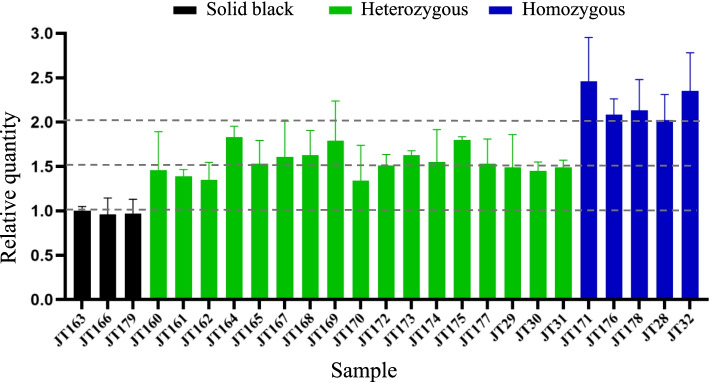
qPCR validation of the 13,420-bp tandem duplication in JT goats. Three sequenced solid black JT goats were selected as a control group. Besides 17 sequenced goats with non-classic Swiss markings, five additional JT goats with beige markings (i.e., JT28, JT29, JT30, JT31, and JT32) were included in qPCR validation

We also did quantitative real-time PCR (qPCR) to examine the copy numbers of the duplicated sequence in the 20 sequenced JT goats and five additional goats with non-classic Swiss markings. The qPCR results agreed with the genotypes obtained from the WGS data, and verified that there were two copies of the duplicated sequence in the JT goats with non-classic Swiss markings (Fig. 4).

We further carried out PCR amplification to validate the presence or absence of the 13,420-bp duplication in 314 goats from JT and six other breeds (JT: *n* = 80, CB: *n* = 20, TC: *n* = 36, Jianchang Black - JC: *n* = 40, Nanjiang Yellow - NJ: *n* = 72, Shaanbei White Cashmere – SC: *n* = 40, Boer: *n* = 26), which included the 94 goats (i.e., JT: *n* = 65, CB: *n* = 15, TC: *n* = 14) that were whole-genome sequenced. The PCR results for the duplication, as expected, showed the presence of the duplication in 22 JT goats with non-classic Swiss markings, whereas the duplication was absent in 281 of the 292 goats from JT and six other populations without the non-classic Swiss markings (Table 1). In summary, the results above indicated that the duplication was a necessary but not sufficient condition for the non-classic Swiss marking phenotype in goats.

**Table 1:** PCR validation for the presence or absence of the 13,420-bp duplication in 314 goats with or without non-classic Swiss markings from seven populations

Population	Coat color	***n***	Presence of the duplication	
			Yes	No
JT	Non-classic Swiss markings	22	22	0
JT	Solid black	58	2	56
CB	Black-and-tan	20	0	20
TC	Solid black	36	4	32
JC	Solid black	40	0	40
NJ	Black-and-tan	72	1	71
SC	Solid white	40	0	40
Boer	White with the pigment on the head	26	4	22

### In silico analysis of the sequence composition and functions of the 13,420-bp duplication

**Fig. 5:**
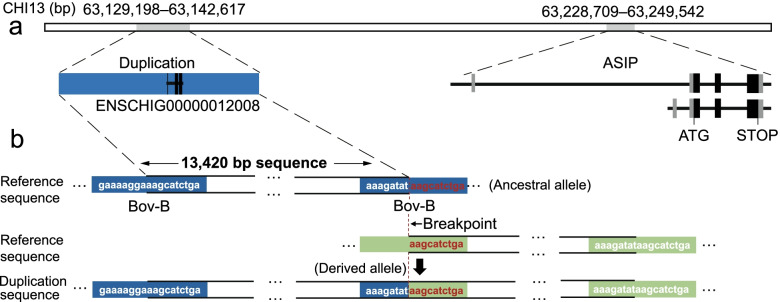
Sequence composition analysis of the 13,420-bp duplication in the major associated region. **a** The genomic locations of the 13,420-bp duplication and the *ASIP* gene (genomic positions refer to the ARS1 assembly). **b** The schematic diagram of the 13,240-bp tandem duplication formation as a result of Bov-B-mediated NAHR

According to genome annotations, the duplication harbors a novel gene (i.e., ENSCHIG00000012008, CHI13:63,136,535–63,140,279; *LOC108637418* in NCBI, also known as *CCDC115* pseudogene) and resides ~ 101 kb upstream of the *ASIP* gene (Fig. 5a and Additional file 4). The online evolutionary conservation analysis (Ruminant Genome database, http://animal.nwsuaf.edu.cn/code/index.php/RGD) of the duplication sequence showed the mean base-wise phastCons and PhyloP scores of 0.43 and 0.32 across 51 Ruminantia species, respectively (Additional file 9).

To better understand the potential function of the duplication, we also examined the types and proportions of repetitive elements in this 13,420-bp sequence. The identified repetitive sequences accounted for 36.78% (4936 bp) of the whole sequence, with the RTE/Bov-B as the largest repeat class followed by LINE-1 (long interspersed element 1) (Additional file 9). We detected the presence of two flanking highly similar RTE/Bov-B elements around the duplication of 13,420-bp sequence (Fig. 5b).

## Discussion

In this study, we observed a simple segregation pattern of the non-classic Swiss markings and solid black coat colors at the individual level in JT goats. Based on the well-established knowledge of pigmentation in animals, the phenotypic difference in solid-black goats and the individuals with non-classic Swiss markings is likely to be from melanin-type switching that is commonly regulated by both *ASIP* and *MC1R* genes. As expected, our GWAS using high-density SNP genotype data from whole-genome sequencing revealed a strong association between a genomic region at the *ASIP* locus on chromosome 13 and the pigmentation phenotype of Swiss markings in goats, which confirm a recent finding based on the detections of selection signatures in two Swiss goat populations [19]. Our result is consistent with the studies in dogs [21] and white wagtails [22] since the similar pigmentation patterns across the body surface (i.e., a black dorsum and yellow/white markings on the head, belly, and legs) in these species.

Identifications of the causal variants at the *ASIP* and *MC1R* loci underlying a specific pigmentation trait remain challenging because there are multiple alleles at both loci within a species (e.g., dogs [21], sheep [5], pigs [23], and chickens [24]). In goats, up to 11 alleles at the *ASIP* locus were proposed to explain different coat color patterns [10], supported by the recent findings of multiple CNVs at this locus [19]. In this study, the genotype segregation of the top associated SNP appeared to agree with a Mendelian mode of inheritance of the coat color (solid back vs. non-classic Swiss markings) in JT goats. However, we excluded the causality of all the SNPs and indels within the strong association signal harboring *ASIP*, based on variant annotations and the genotype distributions in JT goats and other analyzed goat populations.

We then found that a 13,420-bp duplication ~ 101 kb upstream (CHI13: 631,29,198–63,142,617 bp) of *ASIP* was highly associated with the non-classic Swiss marking phenotype in JT goats, which was previously reported as a length of 13,433-bp based on pooled WGS data [19]. This association was supported by the presence of the duplication in three other domestic goat breeds with Swiss markings and its absence in most individuals from four other populations without Swiss markings. The absence of the duplication in modern Bezoars also indicated that this variant might be a de novo derived mutant after domestication. However, our study cannot completely rule out the existence of other unknown genetic loci because several goats without Swiss markings were heterozygotes or homozygous for the duplication allele. In other words, the results from multiple populations demonstrated that the 13,420-bp duplication is necessary but not sufficient for the presence of the Swiss marking depigmentation phenotype in goats. Given the pigmentation variations in the goats with non-classic Swiss markings, we realized that other unknown alleles at the *ASIP* locus or other genetic loci may also influence the tan and beige markings and even points on the face. However, a large sample size is needed to allow further examination. In summary, our findings strongly support that the duplication site has a predominant role for the presence/absence of the pigmentation pattern in goats.

One interesting finding in our study was the changes in the copy number of the candidate duplication between different goat breeds having Swiss markings. Based on the genomic coverage analyses of WGS data, the duplicated sequence replicated only once in the genomes of JT goats with non-classic Swiss markings. In contrast, there seemed to be four or eight copies of the 13,420-bp fragment on chromosome 13 in three European breeds with Swiss markings (i.e., BAL, TOG, and BST goats). Such difference implies that these CNV alleles are unstable in domestic goats. The allele described here for non-classic Swiss marking may arise from the previously published derived allele for Swiss markings [19] or vice versa. Long-read sequencing is needed to gain a deep insight into the complexity at the *ASIP* locus in the goat genome. These results also revealed that the variation in the copy number of the duplication did not lead to heterogeneity in coat color phenotypes in goats, suggesting a threshold effect instead of a simple dose-response effect at the molecular level.

In this study, the 13,420-bp duplicated sequence encompasses several members of the LINE repeat family, such as partial sequences of LINE-1 and Bov-B. The breakpoint flanking repeats have the potential to mediate nonallelic homologous recombination (NAHR) between them and thereby generate recurrent genomic arrangements in the mammalian genome (e.g., human [25]). Given the high similarity of the breakpoint flanking Bov-B elements in both ends of the 13,420-bp sequence, the duplication of this sequence in the goats with non-classic Swiss markings was very likely to be a result of NAHR mediated by these Bov-B repeats. In addition, previous studies demonstrate that transposons may contribute to the phenotypic diversity in animals. For example, the insertion of a 2809-bp LINE-1 sequence into *ASIP* is responsible for the white coat color in water buffaloes [26], and the insertion of LINE-1 into the upstream of *ASIP* also exists in Normande cattle [27]. In domestic dogs, a SINE insertion at the *ASIP* locus is thought to cause the black-and-tan and saddle tan phenotypes [6]. At the transcription level, ventral-specific and hairy-cycle-specific promoters are responsible for the expression of different *ASIP* transcripts in mammals [28–31] and birds [32, 33], respectively. The distribution of white or tan hairs on the body surface of goats with Swiss markings suggested the product of ventral-specific ASIP transcript may underlie this phenotype, similar to the results in dogs [30]. The non-classic Swiss marking phenotype in newborn goat kids can reflect the expression of the ventral-specific *ASIP* transcript during fetal development. Furthermore, the expression of *ASIP* was ~ 40-fold higher in the pheomelanic skin regions than that in the eumelanic skin regions in one BST goat [19]. Given the short physical distance between the duplication and the predicted ventral-specific promoter of the goat *ASIP* gene, we speculate that there is a biological interaction between them in which the duplication acts as an enhancer or other regulatory elements.

## Conclusion

We confirmed that a genomic region harboring the *ASIP* gene is a major locus affecting the Swiss marking phenotype in goats using GWAS. Further analyses provided strong evidence that a 13.42-kb duplication upstream of *ASIP* plays a predominant role in the presence/absence of Swiss markings in goats, although the molecular genetic mechanisms remain unsolved. In addition, the changes in copy number of the duplication across different breeds do not lead to phenotypic heterogeneity in goats.

## Materials and methods

### Animals and whole-genome sequencing

In this study, 20 Jintang Black goats were selected from a breeding farm at Jintang County (Sichuan Province, China), which consisted of 17 goats with non-classic Swiss markings and three goats with solid black coat color (Additional file 1). The animals were released after sampling. Genomic DNA was extracted from blood using TIANmap Genomic DNA Kit (TIANGEN BIOTECH, China). After the quality assessment the integrity and yield, genomic DNA was then sequenced on an Illumina NovaSeq 6000 sequencer (2 × 150 bp) at Novogene (Beijing, China).

We also included short-read WGS data for 45 JT goats (dataset number: PRJNA548681 and PRJNA734084 in NCBI) generated from our previous studies [15, 34]. Collectively, we finally obtained a total of 65 JT goat samples (48 solid black goats vs. 17 black goats with non-classic Swiss markings) for a GWAS. Furthermore, the non-classic Swiss markings type could be further classified into four subclasses according to the colors of markings or points on the face (Additional file 1). Nevertheless, for the sake of simplicity, the 17 goats with markings or points on the face was grouped as one category (i.e., non-classic Swiss markings).

To accurately determine the putative causal variants, this study additionally employed our previously generated WGS data from two other Chinese breeds (i.e., 15 CB goats with black and tan coat color and 14 TC goats with solid black coat color, PRJNA548681) [15]. We also downloaded publicly available WGS data from NCBI for 21 Bezoar ibexes (*Capra aegagrus*; PRJEB3136), 22 goats from two Moroccan breeds (i.e., 14 MD goats with a predominantly black with brown coat color and 8 MN goats with a solid red coat color, PRJEB3134) [11], and 53 goats with Swiss markings from three European breeds (i.e., 5 BAL [PRJEB25062], 24 TOG [PRJNA310684], and 24 BST goats [PRJNA310684]).

### Alignment of short-reads and variant calling and annotations

For the alignment of short reads and short variant calling, we used the bioinformatics pipelines described in our previous work [15]. In brief, high-quality reads were mapped to the goat reference genome (the ARS1 assembly [35]) using BWA [36], followed by the removal of duplicated reads and local realignment around existing Indels and base quality score recalibration using GATK [37]. We first applied GATK (v 4.0.5.2) HaplotypeCaller module to call short variants (i.e., SNPs and Indels) and merged them using CombineGVCFs. We then obtained high-quality short variants by conducting join calling of all gVCF via the GenotypeGVCFs module followed by VariantFiltration.

The final variant dataset was obtained after discarding the variants with minor allele frequency (MAF) < 0.05 and missing genotype > 10% using VCFtools [38]. The biallelic SNPs were finally extracted and were used for the subsequent GWAS analysis. In addition, SnpEff [39] (v4.3) used to annotate SNPs and Indels, which provides a simple assessment of the putatively functional impacts of variants (i.e., high, moderate, low, and modifier impact) for protein-coding genes.

As described in our previous study, the CNVcaller [40] (a read-depth approach) was applied to detect and genotype copy number variation regions (CNVRs) across autosomes. The alignment results of short reads stored in BAM format were visualized with Integrative Genomics Viewer (IGV) [41] to confirm the existence of several CNV loci of interest. The statistical test of the association between biallelic CNVs and the phenotypes was conducted using the ordinary contingency table in R [42] (version 3.6.0). The relative coverage depths of the genomic regions of interest were visualized using the R package ggplot2 [43].

### Analysis of population structure and genome-wide association study

Although there exist relatedness among the 65 JT goat samples, the PCA analysis was performed to estimate the extent of population structure using GCTA [44] as a validation step. We also assessed the population structure by calculating F_ST_ between 48 solid black JT goats and 17 JT animals with non-classic Swiss markings with 10 kb sliding window in VCFtools (command: --fst-window-size 10,000).

We conducted a GWAS using a linear mixed model approach implemented in EMMAX [45], which can effectively account for population structure (i.e., population stratification and relatedness) in the analyzed samples. The genomic inflation factor (i.e., λ) of the test statistics was calculated as the slope of a linear regression between observed and theoretical quantiles in R [42] (version 3.6.0). The genome-wide genomic control was subsequently used to adjust the weak inflation of test statistics from EMMAX. Manhattan plots were drawn with a custom R script.

### PCR and sanger sequencing of one duplication within the major association region

Quantitative PCR (qPCR) was performed to examine the copy numbers of the duplicated sequence in the 20 sequenced goats and five additional goats with non-classic Swiss markings. The *MC1R* gene was selected as the reference gene for the normalization and the primer information for qPCR validation was shown in Additional file 10. All qPCR reactions of 20 μL were amplified in triplicate for each animal using SYBR Green (2x SYBR Premix Ex Taq, Vazyme Biotech Co.,Ltd). qPCR amplification protocol included 95 °C for 30 s; 39 cycles of 95 °C for 10 s, 60 °C for 30 s; and 95 °C for 10 s. The relative copy numbers of the duplicated sequence were calculated by the 2^-ΔΔCt^ method.

For the 13,420-bp duplicated sequence within the major associated region identified using WGS data, we also performed the PCR amplification to verify the presence/absence of the duplication using one primer pair (Dup_F: 5′-TGCCACCCTTTTCTTTCGAT-3′, Dup_R: 5′-CATGCAGTTGTTCTTCGGAC-3′). The pair of Dup_F and Dup_R primers spanning the breakpoint junction between the duplicated copies amplifies a 3677-bp fragment when the 13,420-bp tandem duplication is present. PCR assays were carried out in a final reaction volume of 10 μL containing 5 μL Taq PCR MasterMix II (2×) (TIANGEN BIOTECH), 0.4 μL Dup_F, 0.4 μL Dup_R, 0.8 μL genomic DNA, and 3.4 μL ddH_2_O. PCR amplification protocol began with 95 °C for 3 min; 33 cycles of 95 °C for 30 s, 64 °C for 30 s, and 72 °C for 3 min; and 72 °C for 7 min. The PCR products were resolved by agarose gel electrophoresis. We finally validated the presence or absence of the duplication in 314 sampled goats from JT and six other domestic breeds with or without the Swiss markings (JT: *n* = 80, CB: *n* = 20, TC: *n* = 36, JC: *n* = 40, NJ: *n* = 72, SC: *n* = 40, Boer: *n* = 26), which included the 94 goats that were whole-genome sequenced.

### The sequence composition and function analyses

To examine the potential functions of the 13,420-bp duplication carefully, we carried out the sequence composition and conservation analyses using the web RepeatMasker (http://www.repeatmasker.org/cgi-bin/WEBRepeatMasker) and Ruminant Genome Database (http://animal.nwsuaf.edu.cn/code/index.php/RGD) [46], respectively. We also attempted to predict the ventral promoter of the goat *ASIP* gene based on the ventral promoter sequence of the dog *ASIP* gene [30] by using the pairwise sequence alignment (https://www.ebi.ac.uk/Tools/psa/emboss_needle/).

## Supplementary Information

**Additional file 1: Table S1.** Summary of the short-read sequencing data and mapping statistics for 169 domestic goats and 21 Bezoars included in this study.

**Additional file 2: Figure S1.** PCA of the 65 sampled JT goats based on the identified biallelic SNPs.

**Additional file 3: Table S2.** The list of the genome-wide significantly associated SNPs for the coat color in 65 JT goats.

**Additional file 4: Table S3.** The list of genes within the major association signal on chromosome 13.

**Additional file 5: Table S4.** Summary of genotypes at five associated missense SNPs and one indel in eight domestic goat populations and Bezoars.

**Additional file 6: Table S5.** Summary of genotypes at two CNV sites within the major association signal in eight domestic goat populations and Bezoars.

**Additional file 7: Figure S2.** The visualization of aligned short reads characterizing the 13,420-bp duplication in the genomes of three goats from each of the JT and three European breeds with Swiss markings using IGV.

**Additional file 8: Table S6.** The genome-coverage analysis and predicted copy numbers of the 13,420-bp duplicated sequence in the total 70 goats with Swiss markings from four domestic goat populations.

**Additional file 9: Figure S3.** The analyses of sequence conservation analysis and repetitive elements in the duplicated sequence.

**Additional file 10: Table S7.** The qPCR primer information to validate the copy number of the 13,420-bp sequence.

## Data Availability

The newly generated raw sequencing data for 20 Jintang Black goats for GWAS in this study are available from the NCBI SRA database (accession number: PRJNA797197).

## Abbreviations

BI: Bezoar ibex
CB: Chengdu Brown goats
MD: Moroccan Draa goats
MN: Moroccan Northern goats
NJ: Nanjiang Yellow goats
JC: Jianchang Black goats
JT: Jintang Black goats
TC: Tibetan Cashmere goats
SC: Shaanbei White Cashmere goats
WGS: Whole-genome sequencing
SNP: Single nucleotide polymorphism
Indels: Short insertions and deletions
CNV: Copy number variation
CHI: *Capra hircus* chromosome
LINE: Long interspersed element
NAHR: Nonallelic homologous recombination
