# Supplementary material for: A 13.42-kb tandem duplication at the ASIP locus is strongly associated with the depigmentation phenotype of non-classic Swiss markings in goats
Source: BMC Genomics. 2022 Jun 13;23:437. doi: 10.1186/s12864-022-08672-9 (PMC9190080; doi:10.1186/s12864-022-08672-9)
Supplement: Supplementary file 2 — Additional file 2: Figure S1. PCA of the 65 sampled JT goats based on the identified biallelic SNPs. [file 12864_2022_8672_MOESM2_ESM.pdf]

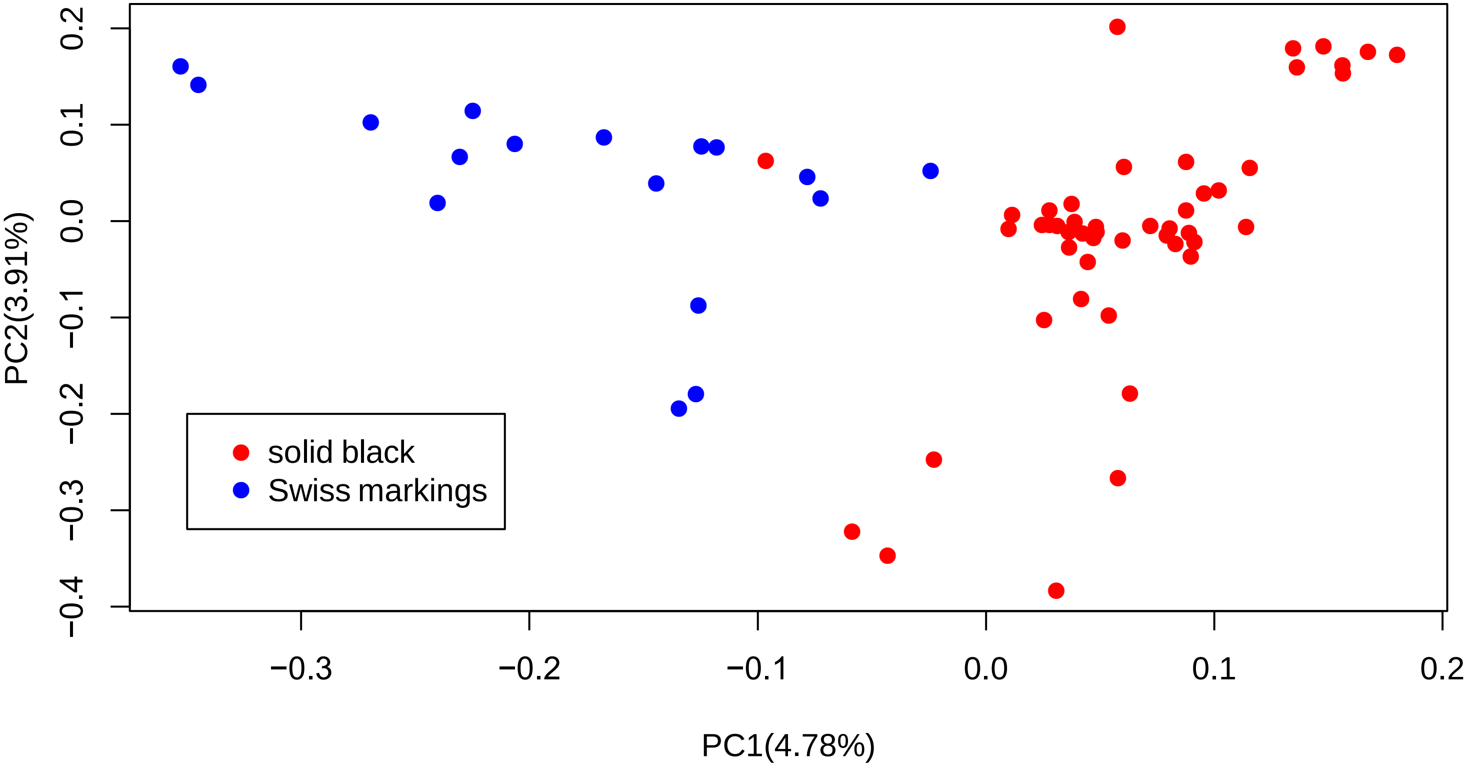

The red and blue circles represented solid black goats and the animals with Swiss markings, respectively.
