# Supplementary figures and images for: A 13.42-kb tandem duplication at the ASIP locus is strongly associated with the depigmentation phenotype of non-classic Swiss markings in goats
Source: BMC Genomics. 2022 Jun 13;23:437. doi: 10.1186/s12864-022-08672-9 (PMC9190080; doi:10.1186/s12864-022-08672-9)

a

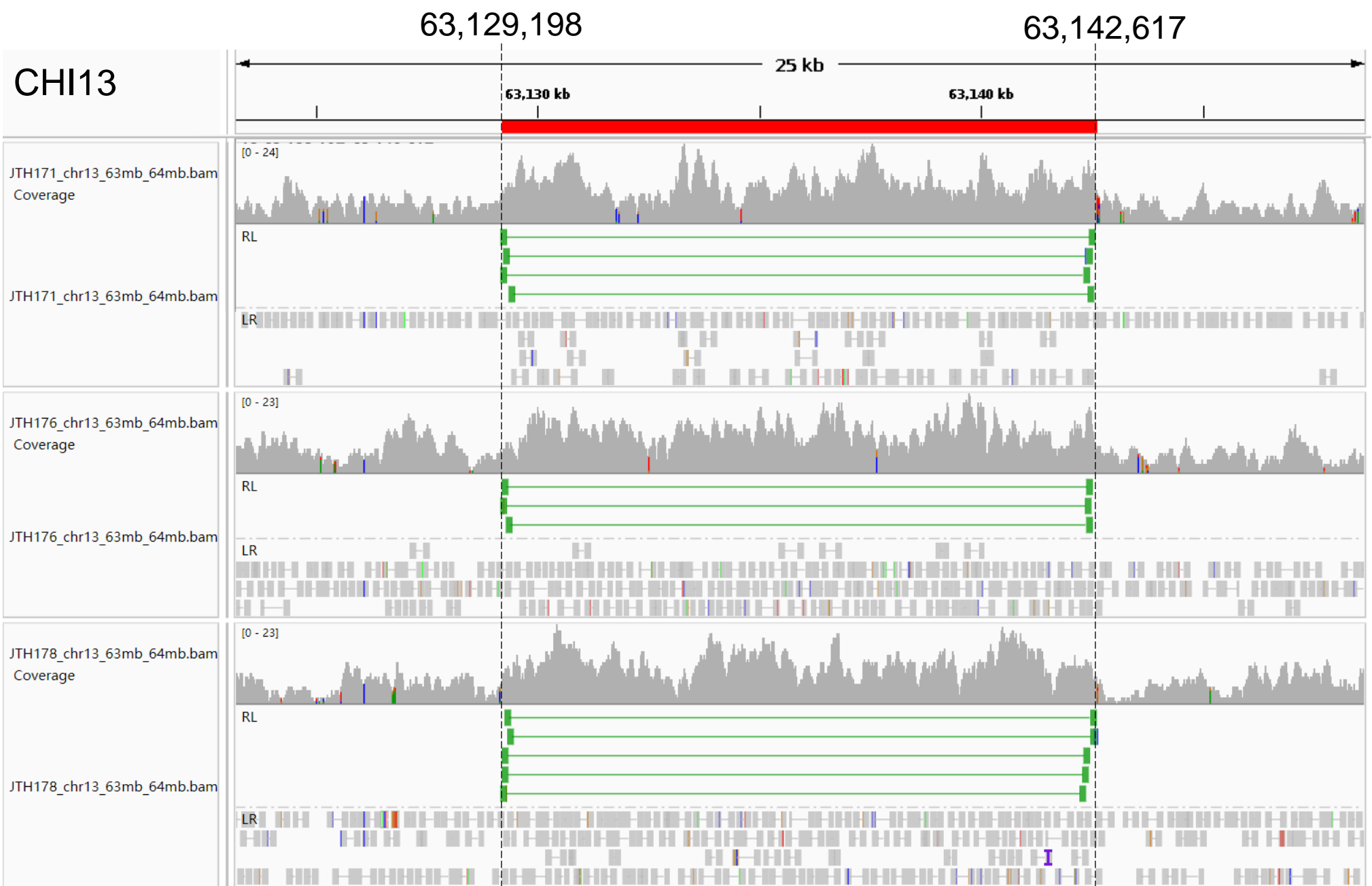

Note: The three JT goats were homozygous for the duplication based on WGS data.

b

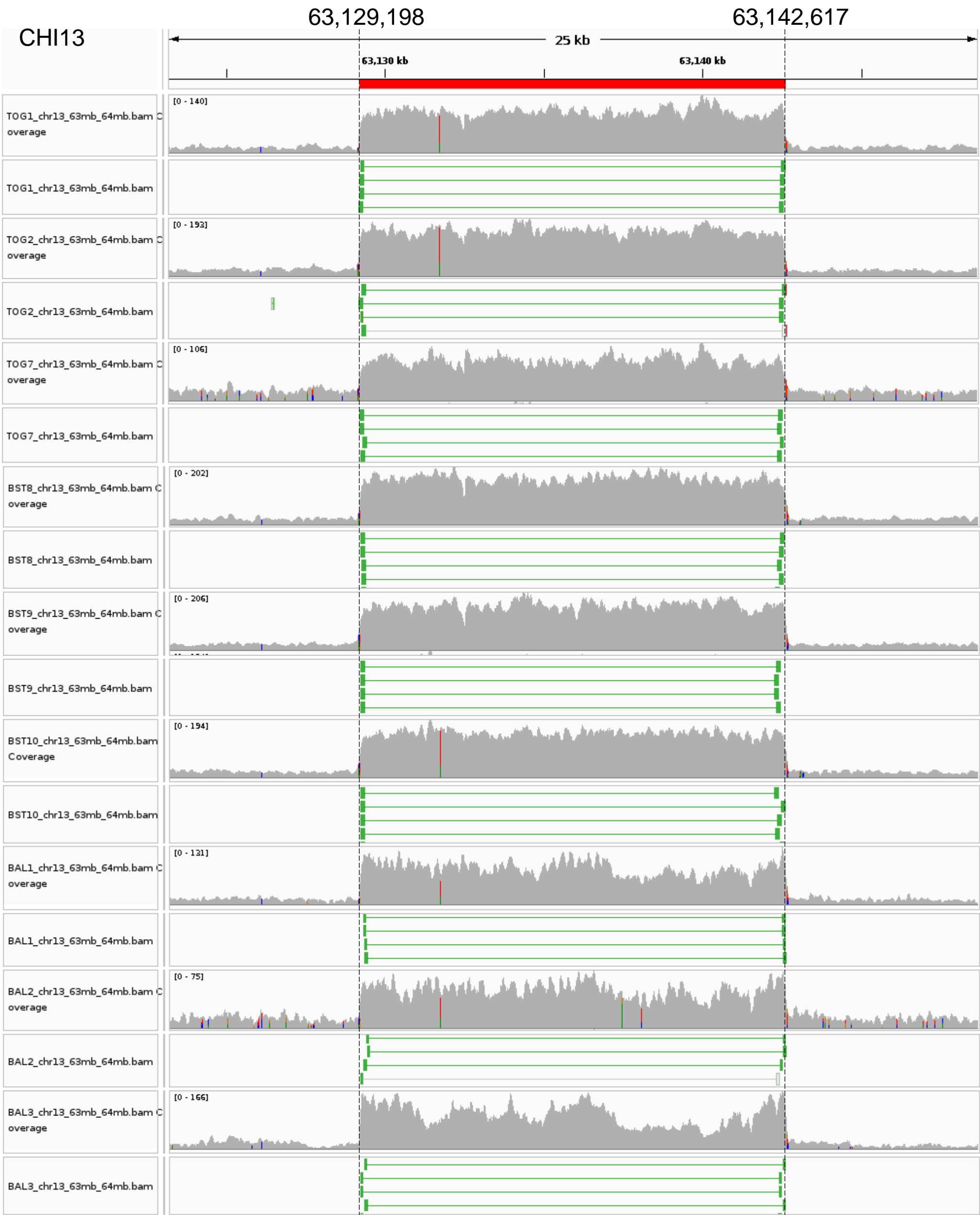

Supplement: Supplementary file 7 — Additional file 7: Figure S2. The visualization of aligned short reads characterizing the 13,420-bp duplication in the genomes of three goats from each of the JT and three European breeds with Swiss markings using IGV. [file 12864_2022_8672_MOESM7_ESM.pdf]
