# Supplementary material for: A 13.42-kb tandem duplication at the ASIP locus is strongly associated with the depigmentation phenotype of non-classic Swiss markings in goats
Source: BMC Genomics. 2022 Jun 13;23:437. doi: 10.1186/s12864-022-08672-9 (PMC9190080; doi:10.1186/s12864-022-08672-9)
Supplement: Supplementary file 9 — Additional file 9: Figure S3. The analyses of sequence conservation analysis and repetitive elements in the duplicated sequence. [file 12864_2022_8672_MOESM9_ESM.pdf]

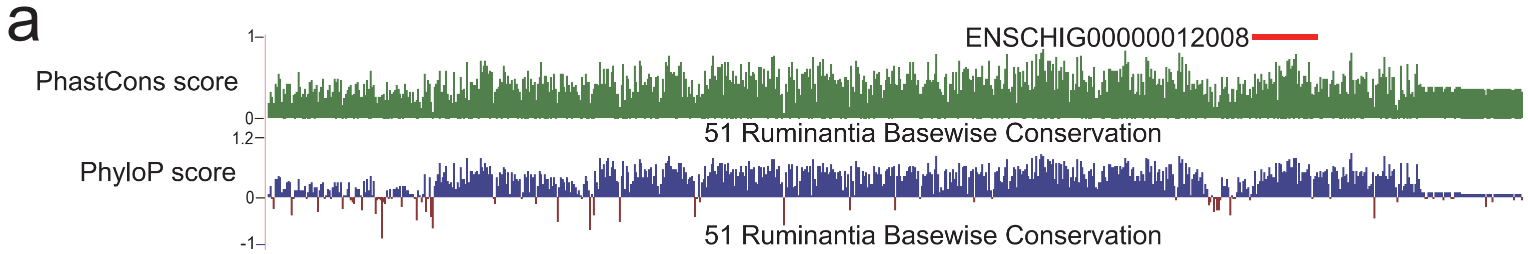

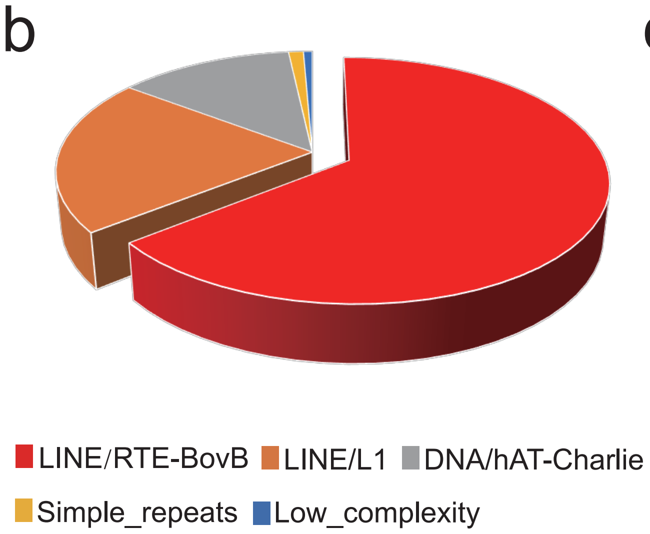

c

Predicted repetitive elements		Matching repeat	Repeat family	SW score
Begin	End			
195	1702	RTE1_ChrAsi	LINE/RTE-BovB	1476
1793	1819	(AACTG)n	Simple_repeat	28
2866	2977	L1M5	LINE/L1	270
2991	3083	L1MA9	LINE/L1	263
3473	3504	A-rich	Low_complexity	12
3582	3726	L1M5	LINE/L1	321
4530	4556	(TCAGT)n	Simple_repeat	14
4787	5419	L1MB7	LINE/L1	2692
5429	5567	MER58A	DNA/hAT-Charlie	513
6988	7661	RTE1_ChrAsi	LINE/RTE-BovB	1291
8876	9099	Charlie1a	DNA/hAT-Charlie	841
9534	9584	L1MC	LINE/L1	309
9574	9859	Charlie1b	DNA/hAT-Charlie	1179
12418	13413	RTE1_ChrAsi	LINE/RTE-BovB	1020

(a) The basewise PhastCons and PhyloP conservation scores of the duplication across 51 Ruminantia species.
(b) Summary of the repetitive elements identified in the duplicated sequence.
(c) The proportions of different classes of the predicted repetitive elements.
