# Supplementary material for: A 13.42-kb tandem duplication at the ASIP locus is strongly associated with the depigmentation phenotype of non-classic Swiss markings in goats
Source: BMC Genomics. 2022 Jun 13;23:437. doi: 10.1186/s12864-022-08672-9 (PMC9190080; doi:10.1186/s12864-022-08672-9)
Supplement: Supplementary file 10 — Additional file 10: Table S7. The qPCR primer information to validate the copy number of the 13,420-bp sequence. [file 12864_2022_8672_MOESM10_ESM.docx]

**Additional file 10 Table S7**

**The qPCR primer information to validate the copy number of the 13420-bp sequence**

| Loci | Primer pairs | Primer sequence | Product size(bp) | Tm (℃) |
| --- | --- | --- | --- | --- |
| 13420-bp sequence | 168_F | CATGGGATTTTCCCTGTGGAAGG | 168 | 60 |
|  | 168_M | CCACACTTAGGGTTTGAGGAAAA |  |  |
| MC1R | 267_F | CTCGTTGGCTTCTTCATAGC | 267 | 60 |
|  | 267_M | GAAGTTCTTGAAGATGCAGCC |  |  |
